# A chemometric approach based on response surface methodology for optimization of antibiotic and organic dyes removal from water samples

**DOI:** 10.1186/s13065-023-01107-w

**Published:** 2024-01-03

**Authors:** Chou-Yi Hsu, Eyhab Ali, Haider Falih Shamikh Al-Saedi, Amjed Qasim Mohammed, Nadia Khalid Mustafa, Maysm Barzan Talib, Usama Kadem Radi, Montather F. Ramadan, Ahmed Ali Ami, Saeb Jasim Al-Shuwaili, Ahmed Alawadi, Ali Alsalamy, Leila Baharinikoo

**Affiliations:** 1 Department of Pharmacy, Chia Nan University of Pharmacy and Science, Tainan City, 71710 Taiwan; 2 Al-Zahraa University for Women, Karbala, Iraq; 3 Faculty of Pharmacy, Department of Pharmaceutics, University of Al-Ameed, Karbala, Iraq; 4 Department of Dentistry, Al-Manara College for Medical Sciences, Maysan, Iraq; 5 Department of Optical Techniques, Al-Noor University College, Nineveh, Iraq; 6 Department of Medical Laboratories Technology, Mazaya University College, Samawah, Iraq; 7 College of Pharmacy, National University of Science and Technology, Dhi Qar, Iraq; 8 College of Dentistry, Al-Ayen University, Thi-Qar, Iraq; 9 Department of Medical Laboratories Technology, Al-Nisour University College, Baghdad, Iraq; 10 Department of Medical Laboratories Technology, Al-Hadi University College, Baghdad, 10011 Iraq; 11 College of Technical Engineering, The Islamic University of Najaf, Najaf, Iraq; 12 College of Technical Engineering, The Islamic University of Al Diwaniyah, Al Diwaniyah, Iraq; 13 College of Technical Engineering, The Islamic University of Babylon, Babylon, Iraq; 14 College of Technical Engineering, Imam Ja’afar Al-Sadiq University, Samawah, Al-Muthanna 66002 Iraq; 15 Department of Analytical Chemistry, Faculty of Chemistry, University of Mazandaran, Babolsar, Iran

**Keywords:** Antibiotic, Central composite design, Dye, Chemometrics

## Abstract

In this study, the Fe_3_O_4_/rGO/Ag magnetic nanocomposite was synthesized and employed as an adsorbent for the removal of tetracycline (TC), crystal violet (CV), and methylene blue (MB) from water samples. The influential parameters in the removal process were identified and optimized using response surface methodology (RSM). Characterization of the product was performed through field emission scanning electron microscopy (FE-SEM), Fourier-transform infrared spectroscopy (FTIR), energy dispersive X-ray spectroscopy (EDX), vibrating-sample magnetometer (VSM), and X-ray diffraction (XRD) analysis. XRD and SEM analysis revealed the successful synthesis of the Fe_3_O_4_/rGO/Ag nanocomposite. EDX analysis elucidated the accuracy and clarity of the chemical composition of the magnetic nanocomposite structure. Additionally, the separation of the nano-adsorbent from the solution can be achieved using a magnetic field. Maximum removal of analytes was obtained at pH of 6, amount of nanocomposite 0.014 g, ultrasonic time of 8 min and concentration of 21 mg L^−1^. Under optimal conditions, the removal efficiencies for TC, CV, and MB were 91.33, 95.82, and 98.19%, respectively. Also, it was observed that after each adsorption–desorption cycle, Fe_3_O_4_/rGO/Ag magnetic nanocomposite had good stability to remove TC, CV, and MB. Achieving nearly 98% removal efficiency in optimal conditions showed that Fe_3_O_4_/rGO/Ag magnetic nanocomposite is an effective adsorbent for removing TC, CV, and MB from wastewater samples.

## Introduction

Access to safe and hygienic drinking water is essential to improve public health [1]. With the rapid growth of urbanization and industrialization of cities, the release of antibiotics and aromatic hydrocarbons in the ecosystem, even at very low concentrations, has increased concerns [2, 3]. Therefore, in order to deal with the contamination of water and living organisms, it is necessary to find ways to remove these substances from the aquatic environment.

Tetracycline (TC) is used as an antibiotic for the treatment of bacterial infections. Over 80% of this substance is excreted from the human body through urine within 2 h of its consumption [4, 5]. Considering the direct biological impact of this chemical compound on microorganisms and the development of antibiotic-resistant bacteria, it poses a significant potential threat [6, 7]. Additionally, the bioaccumulation potential of this compound along the food chain can intensify its toxicity.

The presence of dyes in wastewater creates serious environmental problems due to their high toxicity for aquatic microorganisms and unfavorable aesthetic effects [8, 9]. Crystal violet (CV) and methylene blue (MB) dyes enter the water environment via different sources such as textile industries, printing, fish farming, and cosmetics and hygiene products [10, 11]. Dyes not only give an undesirable color to the water but, in some cases, are harmful compounds that can produce toxic by-products through oxidation, hydrolysis, or other chemical reactions in water [12, 13].

Various methods have been proposed for the removal of antibiotics and dyes, including chemical oxidation, precipitation, distillation, ion exchange, membrane processes, reverse osmosis, and adsorption processes [14–19]. These methods, however, come with challenges and drawbacks such as high costs, low efficiency, prolonged processing times, generation of secondary pollutants, and more [20, 21]. To overcome these issues, research is underway to find more suitable methods, among which surface adsorption processes stand out. The use of surface adsorption processes for purifying different types of wastewaters has been on the rise, holding significant importance in the field of wastewater treatment [22, 23].

The adsorption method can be employed using various materials such as activated carbon, magnetic nanoparticles, modified nanoparticles, modified silica nanoparticles, metal oxide nanoparticles, and carbon nanotubes [24–26]. In the past two decades, extensive research has been dedicated to advancing nanotechnology and nanomaterials. Magnetic nanoparticles have multifaceted applications in various fields, and due to their high surface area, favorable separation characteristics in external magnetic fields, and enhanced adsorption capabilities through surface modification, they have garnered significant attention in the removal of antibiotics and dyes [27, 28]. Among magnetic nanoparticles, metal oxide magnetic nanoparticles are highly regarded for their ease of separation by creating a magnetic field. Additionally, magnetically combined nanoparticles with support matrices facilitate separating and recovering these particles from aqueous solutions [29]. This separation and recovery contribute to reducing the costs of water and wastewater treatment processes. Extensive laboratory studies have shown that iron nanoparticles can be utilized for the removal of dye and antibiotics from contaminated wastewater [30, 31].

Rouhani et al. [32] investigated the removal of tetracycline from water and wastewater samples using the Fe_3_O_4_/Clinoptilolite nanocomposite. The study focused on examining the parameters of contaminant concentration, adsorbent, and pH in a batch system. The maximum efficiency of tetracycline removal by Fe_3_O_4_/Clinoptilolite nanocomposite was 98.6% at pH 7 [32].

In another study, Lin et al. [33] utilized iron oxide nanoparticles synthesized from the extract of *Excoecaria cochinchinensis* leaf as an adsorbent for the removal of Cd (II). The research explored the effects of pH, temperature, adsorbent dosage, and ionic strength. The maximum Cd (II) removal efficiency reached 98.50% under the conditions of an ionic strength of 0.07 M, pH of 8.07, temperature of 45 °C, and an absorbent dosage of 2.5 g L^−1^ [33].

The method of experimental design and analysis of experimental results using response surface methodology (RSM) has been widely employed in many studies. RSM is highly beneficial for designing experiments and analyzing data in a way that enables targeted and reliable conclusions [34]. Essentially, RSM is a specific set of mathematical and statistical methods used for experiment design, model construction, evaluating optimal conditions, and assessing the effects of independent variables on dependent variables [35]. RSM has been applied in various studies concerning removing pollutants such as dyes, heavy metals, and antibiotics from aquatic environments [36, 37].

In the present study, the removal efficiency of TC, CV, and MB by the Fe_3_O_4_/rGO/Ag magnetic nanocomposite was investigated. Additionally, the impact of four factors, namely process time, pH, pollutant concentration, and adsorbent amount, on the adsorption efficiency was examined. Subsequently, experiments were designed using the Response Surface Methodology (RSM) in the Design Expert software, and the optimal conditions for the variables were calculated.

## Materials and methods

### Materials and instrumentation

All chemicals used in the experiments were of laboratory-grade purity and were employed without prior preparation. The utilized chemicals included ethanol, tetracycline, hydrochloric acid, iron(II) chloride tetrahydrate, sodium hydroxide, sodium nitrate, acetonitrile, crystal violet dye, potassium permanganate, graphite, iron(III) chloride hexahydrate, silver nitrate, hydrogen peroxide, sodium borohydride, and methanol, all procured from Merck, Germany. Deionized water was used in all experiments. The pH was determined using a pH meter, and sample agitation was performed with a shaker. Analysis of samples containing antibiotic and dye was carried out using UV–Vis spectrophotometer. The morphology, structure, and composition properties of Fe_3_O_4_/rGO/Ag magnetic nanocomposite were explored using field emission scanning electron microscopy (FE-SEM), Fourier-transform infrared spectroscopy (FTIR), energy dispersive X-ray spectroscopy (EDX), vibrating-sample magnetometer (VSM), and X-ray diffraction (XRD).

### Synthesis of graphene oxide (GO)

Graphene oxide (GO) was synthesized using the modified Hummers method [38]. To achieve this, 1 g of graphite and 1.5 g of sodium nitrate were added to 54 mL of concentrated sulfuric acid and placed in an ice bath. Subsequently, 4 g of potassium permanganate were gradually added to the mixture with vigorous stirring. The ice bath was then removed, and the suspension was allowed to reach a temperature of 35 °C. The solution was held in this state for 45 min. Following this, 150 mL of distilled water was added to the mixture with stirring, and the temperature was increased until the color changed to brown. The mixture was stirred for 24 h at room temperature. Then, 3.5 mL of 30% hydrogen peroxide was gradually added to the solution with stirring to convert the remaining permanganate and manganese dioxide to manganese sulfate. At this stage, a black precipitate formed. The resulting suspension was filtered and washed three times with 3% hydrochloric acid to remove metal impurities. The wash was then continued with water until the pH of the liquid obtained from centrifugation reached approximately 7. To disperse the graphene layers, the final wash of the precipitates was carried out with water for 30 min in an ultrasonic bath. Finally, the precipitate was separated and dried at 40 °C for 24 h.

### Synthesis of Fe_3_O_4_/rGO/Ag magnetic nanocomposite

To synthesize the Fe_3_O_4_/rGO/Ag magnetic nanocomposite, 50 mg of prepared graphene oxide were added to 150 mL of deionized water and sonicated for 2 h. Then, 176 mg of FeCl_3_.6H_2_O and 130 mg of FeCl_2_.4H_2_O were added to the mixture, and the solution’s pH was adjusted to 11 using 1 M NaOH. The temperature of the resulting mixture was raised to 80 °C, and it was stirred for 2 h under these conditions. Subsequently, the obtained precipitate was separated using a magnet, washed several times with deionized water, and then dispersed in 150 mL of deionized water. In the next step, 79 mg of AgNO_3_ were added to the mixture under vigorous stirring. After 15 min, 2 mL of 0.05 M NaBH_4_ solution was slowly added dropwise to the mixture, and the resulting mixture was stirred again at 80 °C for 2 h. The precipitate obtained was then separated by the magnet after reaching room temperature, washed multiple times with deionized water, and finally, the magnetic nanocomposite (Fe_3_O_4_/rGO/Ag) was dried at 60 °C [39].

### ***The pH of the point of zero charge (pH***_***pzc***_***) of adsorbent***

To investigate the removal mechanism of dyes and antibiotics more precisely, the pH of the point of zero charge (pH_pzc_) was determined. The pH_pzc_, where the adsorbent surface is electrically neutral and uncharged. The pH_pzc_ measurement was conducted in 10 flasks containing a solution with 0.1 M NaCl. The initial pH of the solution was adjusted from 1 to 10 using 0.01 M NaOH and 0.01 M HCl. Subsequently, 0.1 g of the adsorbent material was added to each solution, and the electrolyte solution was stirred with the adsorbent for 24 h. After reaching equilibrium, the pH of the solutions was measured. The ∆pH versus initial pH curve was plotted, and the pH_pzc_ value was estimated.

### Experiment design

Various parameters play a role in the surface adsorption process. Therefore, optimizing these parameters is crucial for achieving a high surface adsorption rate of analytes. Among the different protocols and methods available, RSM has gained strength in recent years as the most efficient statistical technique for analyzing and optimizing the parameters of various processes. RSM includes a set of statistical and mathematical techniques to build an experimental model and its purpose is to optimize the response by carefully designing experiments to simultaneously understand the interactive effects between variables [40]. This analysis begins with designing a series of experiments to obtain sufficient predictions of a response. Then, fitting a hypothetical (empirical) model to the data obtained in the selected design and finally determining the optimal conditions on the input variables of the model, leading to maximizing or minimizing the study’s response, can determine the impact of different factors on the effectiveness of the result [41]. Furthermore, by examining parametric effects and interactions, it can identify a combination of factors and values needed to maximize effectiveness (pollutant removal). The design and optimization of reaction parameters were conducted using RSM testing and the Design Expert v.12 software.

In this regard, effective parameters in the removal of pollutants during the surface adsorption process, including time, the amount of nanocomposite, analyte concentration, and pH, were investigated using the Box–Behnken Design (BBD) method. This method not only predicts reliable results as a function of other variables but also introduces the best mathematical model. Other advantages include estimating the second-degree model parameters, and design points, the need for fewer experiments, creating sequential designs, detecting the lack of fit in the model, and utilizing blocks. The impact of these four studied variables, their ranges, and coded levels are also shown in Table 1. Based on this, a second-order model is fitted to predict the optimal point for determining the correlation between independent variables and responses better. For these four variables, the prediction model is expressed as Eq. 1.

1 $$ {\text{Y}}\, = \,\beta_{0} + \,\sum\nolimits_{i = 1}^{k} {\beta_{i} X_{i} } \, + \,\sum\nolimits_{i = 1}^{k} {\beta_{ii} X_{i}^{2} } \, + \,\sum\nolimits_{i \le j}^{k} {\sum\nolimits_{j}^{k} {\beta_{ij} X_{i} } } X_{j} \, + \,e $$

**Table 1:** The design matrix

Variables	Symbol	Unit	Range and levels		
			− 1	0	+ 1
pH of the solution	A	–	4	7	10
Analyte concentration	B	mg L^−1^	10	20	30
Nanocomposite amount	C	g	0.005	0.010	0.015
Ultrasound time	D	min	5	10	15

In this equation, *Y* represents the calculated response, *β*_*0*_ is the model constant coefficient, *X*_*i*_ and *X*_*j*_ are independent variables, *β*_*i*_ and *β*_*j*_ are linear coefficients, *β*_*ij*_ is the interaction coefficient, and *β*_*ii*_ is the second-degree coefficient.

### Experimental procedure

In the current study, initial experiments were conducted on laboratory samples, and ultimately, after obtaining the optimal conditions, experiments were performed on industrial wastewater. For each trial, 50 mL of prepared solutions with different concentrations were poured into Erlens. Then, 0.01 M HCl and 0.01 M NaOH solutions were used to adjust the desired pH. In the next step, 0.014 g of the adsorbent was weighed and added to the solution. Subsequently, containers containing the samples were placed in an ultrasonic bath for a specific period. After the desired time, the Erlens were removed from the ultrasonic bath and placed next to a magnet. After the magnetic nanoparticles were absorbed by the magnet and separated from the aqueous solution, an appropriate amount of the sample was poured into a sample container. In the next step, the remaining concentration of dye and antibiotics was analyzed using a UV–Vis spectrophotometer. The removal efficiency was calculated using Eq. 2.

2 $$\%R =\frac{{C}_{0}-{C}_{e}}{{C}_{0}} \times 100$$

where %*R* is the percentage of analyte removal by the Fe_3_O_4_/rGO/Ag magnetic nanocomposite, *C*_*0*_ is the initial concentration, and *C*_*e*_ is the final concentration.

## Results and discussion

### ***Characterization of the Fe***_***3***_***O***_***4***_***/rGO/Ag nanocomposite***

**Fig. 1:**
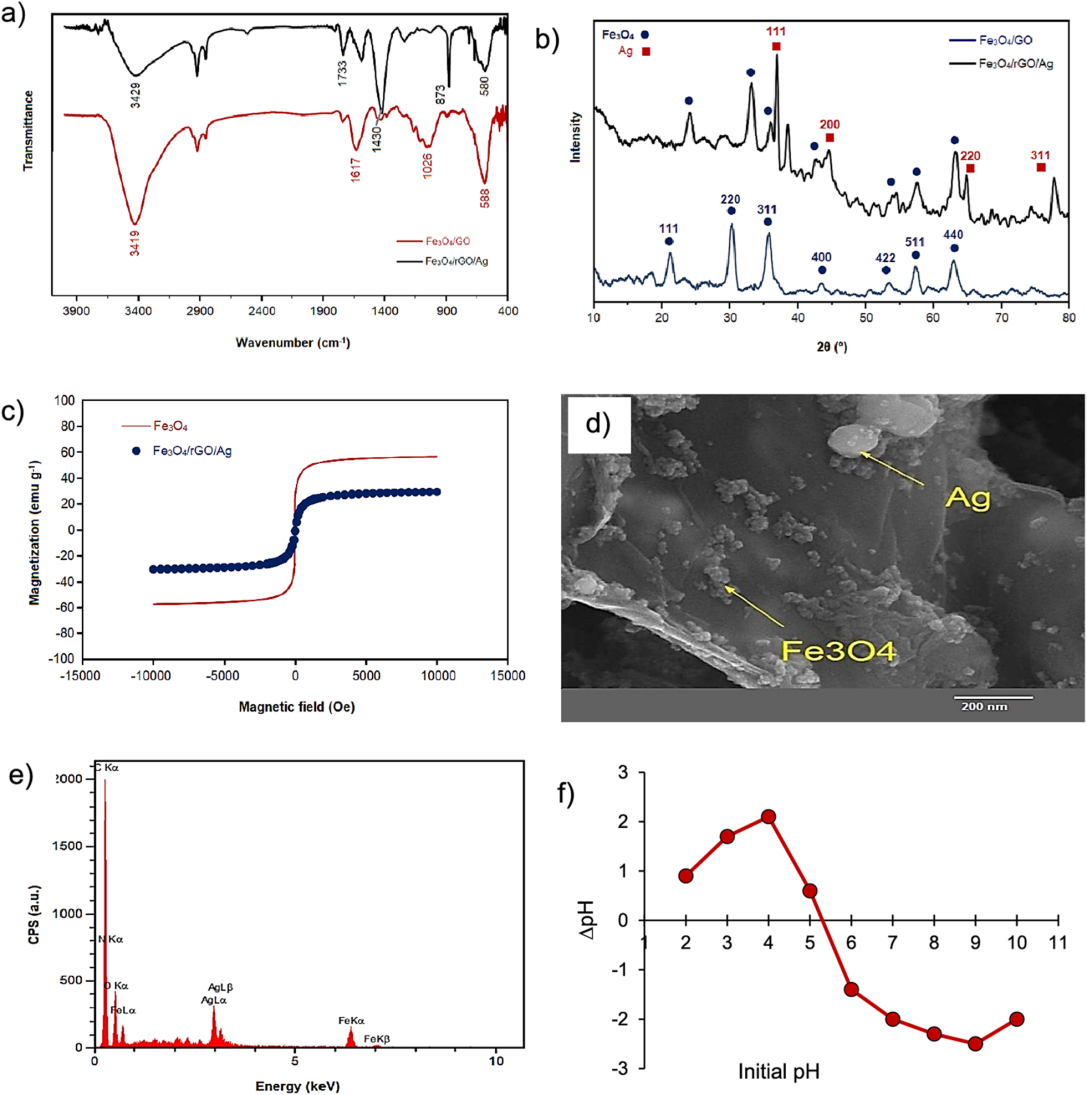
**a** FTIR, **b** XRD, **c** VSM, **d** SEM **e** EDX, and **f** pH_pzc_ of Fe_3_O_4_/rGO/Ag nanocomposite

FT-IR spectroscopy was employed to determine the nature and confirm the presence of functional groups in the structure of the materials. The FT-IR spectra of magnetic graphene oxide (Fe_3_O_4_/GO) and magnetic nanocomposite Fe_3_O_4_/rGO/Ag are shown in Fig. 1a. In both spectra, a broad peak at 3419 cm^−1^ corresponds to the stretching mode of hydroxyl groups present in the graphene layers. The peaks at 1730^1^, 1617, 1035, 1430, and 1260 cm^−1^ represent the stretching modes of the C=O group in carboxyl, the C=C group, the C-O-C group, the C-OH group, and the C-O vibration in the epoxy group, respectively. Additionally, a peak observed in the region of 580 cm^−1^ corresponds to the stretching mode of the Fe-O bond.

Figure 1b illustrates the X-ray diffraction (XRD) patterns of reduced graphene oxide (rGO) and magnetic nanocomposite Fe/rGO/Ag in the 2θ range of 10–80°. The diffraction patterns in Fig. 1b exhibit four peaks at 37.89, 44.25, 64.43, and 78.61°, corresponding to the crystalline surfaces (111), (200), (220), and (311), respectively, which are characteristic of the crystal structure of silver nanoparticles. This diffraction pattern aligns well with reported data (JCPDS no. 0783–04) [42]. The XRD pattern related to magnetic iron oxide nanoparticles shows seven peaks at 21.97, 30.18, 35.64, 42.83, 54.51, 57.15, and 63.09°, confirming the crystalline surfaces (111), (220), (311), (400), (422), (511), and (440) of these nanoparticles. This observation is in good agreement with patterns reported in the literature [43]. The XRD pattern of the synthesized nanocomposite clearly displays all peaks corresponding to the structures of silver nanoparticles and magnetic nanoparticles.

The magnetic behavior of Fe_3_O_4_ and Fe_3_O_4_/rGO/Ag was measured under an applied magnetic field ranging from 10 to 10 kOe at room temperature. According to the results presented in Fig. 1c, the maximum magnetic saturation of Fe_3_O_4_ nanoparticles and Fe_3_O_4_/rGO/Ag nanocomposite is 57.5 and 30.1 emu g^−1^, respectively. As the results indicate, the addition of silver nanoparticles and graphene sheets to the nanocomposite reduces the magnetic saturation of Fe_3_O_4_ nanoparticles. However, this level of magnetic saturation is sufficient for separating the nano-adsorbent from the solution using a magnet.

The FE-SEM image of the Fe_3_O_4_/rGO/Ag magnetic nanocomposite is provided in Fig. 1d. The structure of this nanocomposite appears as reduced graphene oxide sheets with magnetic Fe_3_O_4_ and silver nanoparticles forming accumulations of spherical and quasi-spherical nanoparticles with an average size below 50 nm on its surface. The silver nanoparticles, depicted with a brighter color and larger dimensions than the dark-colored magnetic Fe_3_O_4_ nanoparticles, exhibit a clustered and semi-aggregated morphology in the images.

EDX has been utilized for elemental analysis and precise determination of the chemical composition of the Fe_3_O_4_/rGO/Ag magnetic nanocomposite. The results are presented in Table 2 and Fig. 1e. As observed, the elemental analysis accurately elucidates the presumed chemical composition for the Fe_3_O_4_/rGO/Ag magnetic nanocomposite structure.

**Table 2:** Results of elemental analysis of Fe_3_O_4_/rGO/Ag magnetic nanocomposite

Element	%C	%N	%O	%Fe	%Ag
Fe_3_O_4_/rGO/Ag	59.90	4.37	23.22	5.90	6.62

Also, the pH of Fe_3_O_4_/rGO/Ag adsorbent was studied and the obtained results can be seen in Fig. 1f. According to the results shown in Fig. 1f, the pH_pzc_ for the Fe_3_O_4_/rGO/Ag adsorbent was found to be 5.3. Therefore, the surface of Fe_3_O_4_/rGO/Ag adsorbent at pH values lower than and higher than 5.3 has a positive surface charge and a negative surface charge, respectively.

### Investigation of the efficiency of adsorbents in TC, CV, and MB removal

**Fig. 2:**
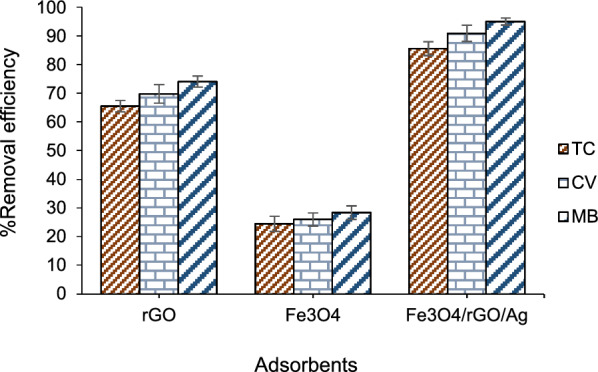
Efficiency of adsorbent type in removing TC, CV, and MB

After preparing the nanocomposite and identifying its characteristics, the efficiency of various adsorbents, including rGO, Fe_3_O_4_, and Fe_3_O_4_/rGO/Ag, was investigated for the removal of TC, CV, and MB. The results obtained are reported in Fig. 2. According to the results, rGO possesses the necessary capability for removing TC, CV, and MB from water samples. However, for better dispersion of graphene sheets and the use of a magnet for easy and rapid separation of the adsorbent from the solution, magnetic nanoparticles were employed. Therefore, among the examined adsorbents, Fe_3_O_4_/rGO/Ag was selected for further studies.

### Optimization of TC, CV, and MB removal conditions using the BBD method

To achieve the best conditions for the removal of TC, CV, and MB, effective parameters in the process were studied using the Box–Behnken Design (BBD) method. As mentioned earlier, four factors, including time (min), adsorbent dosage (g), analyte concentration (mg L^−1^), and pH, were selected. A total of 29 experiments were designed using the Design Expert v.12 software based on the BBD method. According to the design, experiments were conducted by the software to find the optimal range of variables, and the results of TC, CV, and MB removal percentages are presented in Table 3.

**Table 3:** The results of BBD

	Variables				%R-TC		%R-CV		%R-MB	
Run	A	B	C	D	Experimental	Predicted	Experimental	Predicted	Experimental	Predicted
1	7	10	0.005	10	71.02	70.71	72.44	73.04	78.69	78.27
2	7	20	0.015	15	55.25	55.67	70.11	70.13	66.5	67.13
3	4	30	0.010	10	29.88	29.58	50.21	50.87	68.71	70.42
4	7	30	0.015	10	73.59	74.02	82.24	81.36	96.6	96.01
5	10	20	0.005	10	86.62	86.45	78.7	78.92	89.51	90.56
6	7	20	0.005	15	63.74	64.27	59.62	60.35	68.67	70.30
7	7	30	0.005	10	35.14	34.85	49.37	48.35	61.27	59.63
8	4	20	0.005	10	19.17	19.90	37.4	36.78	42.28	41.20
9	7	20	0.010	10	84.42	83.32	88.75	89.46	95.79	93.48
10	4	20	0.010	5	35.19	35.64	62.82	63.45	64.6	64.62
11	10	10	0.010	10	49.13	49.37	63.52	62.97	70.44	69.81
12	10	20	0.015	10	43.79	43.00	56.39	57.19	57.33	58.34
13	7	20	0.010	10	82.44	83.32	90.34	89.46	94.15	93.48
14	7	20	0.005	5	58.32	57.84	67.18	67.27	67.27	67.72
15	10	20	0.010	5	54.03	54.53	63.61	63.17	68.82	67.06
16	10	30	0.010	10	32.13	32.69	52.88	53.76	59.46	60.82
17	4	20	0.010	15	29.65	29.26	45.45	45.61	48.58	49.33
18	7	20	0.010	10	83.15	83.32	90.18	89.46	93.49	93.48
19	7	20	0.015	5	79.54	78.95	85.07	84.44	88.91	88.36
20	7	20	0.010	10	82.33	83.32	89.83	89.46	92.71	93.48
21	7	30	0.010	5	55.35	55.24	67.36	67.63	77.39	77.92
22	7	10	0.010	10	49.7	49.76	62.27	62.18	60.17	59.57
23	7	10	0.010	5	33.92	34.15	64.59	64.68	58.59	59.89
24	7	10	0.015	10	43.63	44.04	66.25	66.99	58.71	59.34
25	7	30	0.010	15	23.08	22.79	48.81	48.90	60.96	59.59
26	10	20	0.010	15	44.41	44.07	60.68	59.77	64.74	63.70
27	7	20	0.010	10	84.26	83.32	88.21	89.46	91.26	93.48
28	4	20	0.015	10	75.73	75.85	85.5	85.46	92.01	90.89
29	4	10	0.010	10	19.39	18.77	52.75	51.97	43.68	43.40

ANOVA was employed to assess the significance and validity of second-degree models that were predicted, and the Fisher statistical test was used to examine the influence of factors on the response variables. The software determined the importance and impact of the estimated coefficients for each variable and all possible interactions between them on the response variables. To identify important factors and create a model for optimization, a second-degree model incorporating all terms in Eq. 1 was utilized in the experimental design.

Effects with less importance than 95% or, in other words, effects with p-values greater than 0.05 (p > 0.05) were considered errors and were eliminated [44]. Subsequently, a new analysis of variance was performed for the reduced model. Replicating central points (n = 5) in the experimental design was done to estimate the amount of experimental error. On the other hand, the coefficients of determination (R^2^), adjusted R^2^ (Adj-R^2^), and predicted R^2^ (Pred-R^2^) can serve as quick and easy tools to assess the model's conformity to the predictive power, especially when removing one of the data. These three parameters should not differ significantly for an appropriate model. Some features of the reduced model obtained for TC, CV, and MB are observable in Tables 4, 5, 6, respectively.

**Table 4:** ANOVA for removal of TC

Source	Sum of squares	DF*	Mean square	F-value	p-value	
Model	13746.47	14	981.89	1643.04	< 0.0001	Significant
A	851.77	1	851.77	1425.30	< 0.0001	Significant
B	25.87	1	25.87	43.29	< 0.0001	Significant
C	117.31	1	117.31	196.30	< 0.0001	Significant
D	212.69	1	212.69	355.90	< 0.0001	Significant
AB	188.93	1	188.93	316.14	< 0.0001	Significant
AC	2469.59	1	2469.59	4132.49	< 0.0001	Significant
AD	4.16	1	4.16	6.96	0.0194	Not significant
BC	1083.73	1	1083.73	1813.45	< 0.0001	Significant
BD	577.20	1	577.20	965.86	< 0.0001	Significant
CD	220.67	1	220.67	369.26	< 0.0001	Significant
A^2^	4106.66	1	4106.66	6871.86	< 0.0001	Significant
B^2^	4236.60	1	4236.60	7089.31	< 0.0001	Significant
C^2^	22.42	1	22.42	37.52	< 0.0001	Significant
D^2^	1936.67	1	1936.67	3240.72	< 0.0001	Significant
Residual	8.37	14	0.5976			
*Lack of Fit*	4.49	*10*	0.4489	*0.4632*	*0.8524*	Not significant
*Pure Error*	3.88	*4*	0.9693			
Cor Total	13754.84	28				
R^2^ = 0.9994	Adjusted R^2^ = 0.9988		Predicted R^2^ = 0.9977		Adeq-Precision = 121.71	

^*^Degree of freedom

**Table 5:** ANOVA for removal of CV

Source	Sum of Squares	DF*	Mean Square	F-value	p-value	
Model	6531.94	14	466.57	549.86	< 0.0001	Significant
A	144.56	1	144.56	170.37	< 0.0001	Significant
B	79.83	1	79.83	94.08	< 0.0001	Significant
C	544.73	1	544.73	641.98	< 0.0001	Significant
D	338.03	1	338.03	398.38	< 0.0001	Significant
AB	16.40	1	16.40	19.33	< 0.0001	Significant
AC	1239.39	1	1239.39	1460.66	< 0.0001	Significant
AD	52.13	1	52.13	61.43	0.0006	Not significant
BC	381.42	1	381.42	449.52	< 0.0001	Significant
BD	65.85	1	65.85	77.61	< 0.0001	Significant
CD	13.69	1	13.69	16.13	0.0013	Not significant
A^2^	2270.63	1	2270.63	2676.00	< 0.0001	Significant
B^2^	1632.07	1	1632.07	1923.44	< 0.0001	Significant
C^2^	246.51	1	246.51	290.52	< 0.0001	Significant
D^2^	1054.83	1	1054.83	1243.15	< 0.0001	Significant
Residual	11.88	14	0.8485			
*Lack of Fit*	8.38	*10*	0.8383	*0.9591*	*0.5675*	Not significant
*Pure Error*	3.50	*4*	0.8741			
Cor Total	6543.82	28				
R^2^ = 0.9982	Adjusted R^2^ = 0.9964		Predicted R^2^ = 0.9918		Adeq-Precision = 79.52	

^*^Degree of freedom

**Table 6:** ANOVA for removal of MB

Source	Sum of squares	DF*	Mean square	F-value	p-value	
Model	7485.59	14	534.69	200.93	< 0.0001	Significant
A	212.02	1	212.02	79.67	< 0.0001	Significant
B	243.99	1	243.99	91.69	< 0.0001	Significant
C	228.55	1	228.55	85.89	< 0.0001	Significant
D	260.96	1	260.96	98.07	< 0.0001	Significant
AB	324.18	1	324.18	121.82	< 0.0001	Significant
AC	1677.31	1	1677.31	630.32	< 0.0001	Significant
AD	35.64	1	35.64	13.39	0.0026	Not significant
BC	764.80	1	764.80	287.40	< 0.0001	Significant
BD	81.09	1	81.09	30.47	< 0.0001	Significant
CD	141.73	1	141.73	53.26	0.0013	Not significant
A^2^	2035.69	1	2035.69	764.99	< 0.0001	Significant
B^2^	1392.46	1	1392.46	523.27	< 0.0001	Significant
C^2^	197.41	1	197.41	74.18	< 0.0001	Significant
D^2^	1379.90	1	1379.90	518.55	< 0.0001	Significant
Residual	37.25	14	2.66			
*Lack of Fit*	25.95	*10*	2.59	*0.9180*	*0.5873*	Not significant
*Pure Error*	11.31	*4*	2.83			
Cor Total	7522.85	28				
R^2^ = 0.9950	Adjusted R^2^ = 0.9901		Predicted R^2^ = 0.9778		Adeq-Precision = 46.71	

^*^Degree of freedom

Regression analysis of the model equations indicates that the significant parameters and their interactions are highly meaningful (p-value < 0.0001). The Prob˃F values identified first-order effects, square effects, and interactions between variables as crucial model terms. The F-values of the model are 1643.04, 549.86, and 200.93 for TC, CV, and MB, respectively. Moreover, the p-value < 0.0001 signifies the significance of the models. The responses of TC, CV, and MB removal process after removing ineffective terms were predicted at a 95% confidence level by Eqs. 3–5.

3 $$ \% {\text{R}} - {\text{TC }} = \, + {83}.{32 } + {8}.{\text{425A }} - {1}.{\text{46B }} + {3}.{\text{12C }} - {4}.{\text{21D }} - {6}.{\text{87AB }} - {24}.{\text{84AC }} - {1}.0{\text{2AD 16}}.{\text{46BC }} - {12}.0{\text{1BD }} - {7}.{\text{42CD }} - {25}.{\text{16A}}^{2} \, - {25}.{\text{55B}}^{2} \, - {1}.{\text{85C}}^{2} \, - {17}.{\text{27D}}^{2} $$

4 $$ \% {\text{R}} - {\text{CV}} = \, + {89}.{462 } + {3}.{\text{47A }} - {2}.{\text{57B }} + {6}.{\text{73C }} - {5}.{3}0{\text{D }} - {2}.0{\text{2AB }} - {17}.{6}0{\text{AC }} + {3}.{\text{61AD }} + {9}.{\text{76BC }} - {4}.0{\text{5BD }} - {1}.{\text{85CD }} - {18}.{7}0{\text{A}}^{2} \, - {15}.{\text{86B}}^{2} \, - {6}.{\text{16C}}^{2} \, - {12}.{\text{75D}}^{2} $$

5 $$ \% {\text{R}} - {\text{MB}} = \, + {93}.{48} \, + {4}.{2}0{\text{A }} + {4}.{5}0{\text{B }} + {4}.{\text{36C }} - {4}.{\text{66D }} - {9}.00{\text{AB }} - {2}0.{\text{47AC }} + {2}.{\text{98AD }} + {13}.{\text{82BC }} - {4}.{5}0{\text{BD }} - {5}.{\text{95CD }} - {17}.{\text{71A}}^{2} \, - {14}.{\text{65B}}^{2} \, - {5}.{\text{51C}}^{2} \, - {14}.{\text{58D}}^{2} $$

**Fig. 3:**
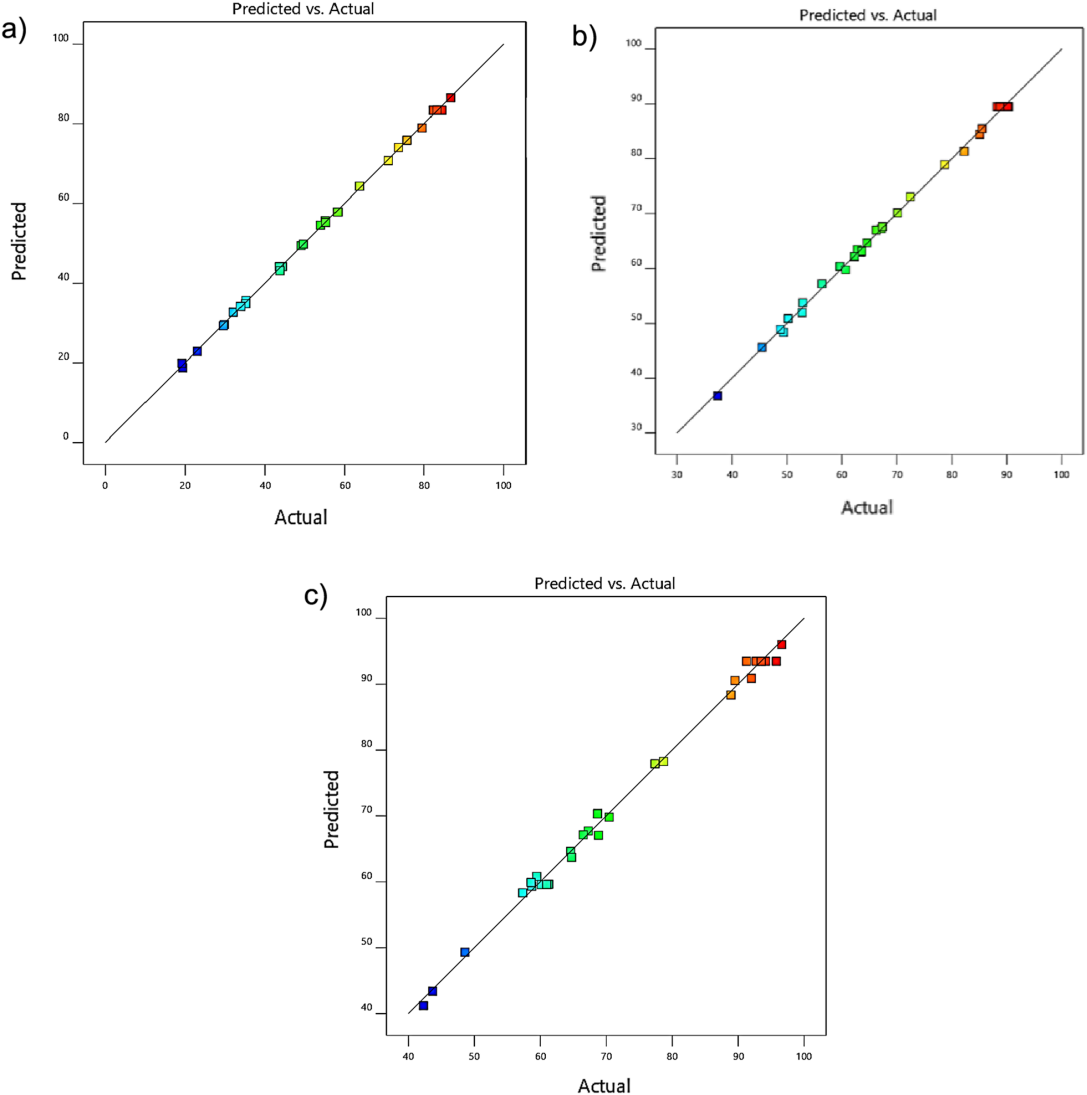
Plot of predicted values versus actual values for **a** TC, **b** CV, and **c** MB

According to the ANOVA results, the R2 values for TC, CV, and MB are 0.9994, 0.9982, and 0.9950, respectively. These high R2 values ensure a satisfactory fit of the models to the experimental data. Furthermore, the Lack of Fit (LOF) corresponding to the F-value of the model (0.4632, 0.9591, and 0.9180 for TC, CV, and MB) indicates that the data variability around the predicted model is not significantly meaningful compared to the pure error. The efficiency and accuracy of the model, with R^2^ values exceeding 0.99, demonstrate excellent agreement between actual and predicted values for TC, CV, and MB removal, as illustrated in Fig. 3a–c using the Fe_3_O_4_/rGO/Ag nanocomposite.

### 3D response surface analysis

**Fig. 4:**
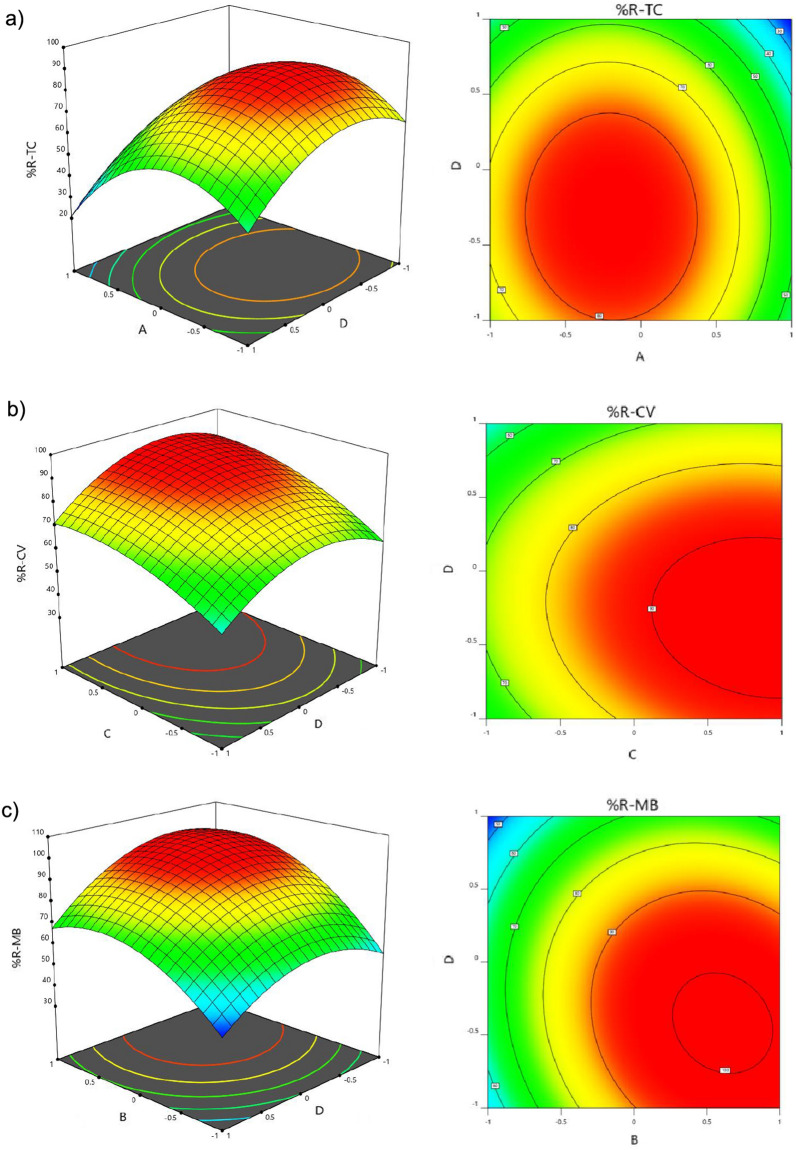
The three-dimensional (3D) plots of removal of **a** TC, **b** CV, and **c** MB

Three-dimensional (3D) plots depicting the predicted responses of the model are presented in Fig. 4a–c, aiming to achieve a surface response for each variable. The response surface plots illustrate the correlation and interaction between two variables and the extent of TC, CV, and MB removal while keeping other variables at central levels. The results reveal a nonlinear relationship between the response and the four variables, demonstrating the dependence of the removal percentage on all examined variables. Optimal conditions were determined based on the response surface plots.

Considering the significant influence of the solution pH in the removal process, this factor was investigated concerning the removal of TC, CV, and MB. The effect of pH on the removal process of TC, CV, and MB was examined within the pH range of 4–10. The obtained results (Fig. 4a) indicate that the adsorption behavior on the surface of the Fe_3_O_4_/rGO/Ag nanocomposite is influenced by the solution pH. Additionally, the pH_pzc_ (point of zero charge) of the Fe_3_O_4_/rGO/Ag nanocomposite was determined to be 5.3. Consequently, the nano-adsorbent surface possesses a positive surface charge at pH values below 5.3 and a negative charge at pH values above 5.3. Increasing the pH beyond the pH_pzc_ and the concentration of hydroxide ions in the solution leads to the creation of a negative charge on the adsorbent surface. In the presence of cations in the solution, electrostatic attraction between the negative charge of the adsorbent surface and the positive charge of cations occurs, enhancing the adsorption of cations from the solution by the adsorbent. Therefore, in the subsequent stages of the research, the optimum pH for the adsorption of analytes onto the Fe_3_O_4_/rGO/Ag adsorbent was determined to be 6.

The impact of CV concentration on the efficiency of surface adsorption removal was investigated by varying the initial concentration from 10 to 30 mg L^−1^ while keeping other parameters constant. As illustrated in Fig. 4b, the removal efficiency of the dye by the employed adsorbent decreases with the increase in dye concentration, which is a natural occurrence. This reduction can be attributed to the fact that, as the dye concentration rises, its remaining amount also increases. Consequently, the removal efficiency decreases, and another contributing factor is the saturation of the adsorbent surface at higher dye concentrations.

Furthermore, based on the results presented in Fig. 4c, it is evident that the MB removal efficiency distinctly increases as a quadratic function with the rise in the adsorbent amount and contact time. This observation can be attributed to the increased availability of active sites for dye interactions due to the elevated amount of the nano-adsorbent. Additionally, the extended contact time provides the necessary opportunity for the adsorption of the analyte by the nano-adsorbent. Therefore, both factors contribute to an increase in the percentage of dye removal. The highest removal efficiency of MB, 98.19%, was achieved under optimal conditions, including a contact time of 8 min and an adsorbent amount of 0.014 g.

### Optimization

The optimization of the model and determination of optimal variable values in the TC, CV, and MB removal process by Fe_3_O_4_/rGO/Ag magnetic nanoparticles was carried out using the software. Essentially, the software determined the optimization conditions for each variable and the corresponding response. To achieve this, all parameters were set within the design range, maximizing the removal efficiency. Experiments were conducted under specified conditions (concentration of 21 mg/L, pH 6, time of 8 min, and a nanocomposite amount of 0.014 g) as outlined in Table 7. The experimental removal efficiencies under these optimal conditions for TC, CV, and MB were 91.33, 95.82, and 98.19%, respectively. Additionally, with the assistance of Design Expert software, the removal percentages were predicted under optimal conditions, resulting in 88.16, 93.25, and 98.41% removal for TC, CV, and MB, respectively.

**Table 7:** Optimum variables of removal of pollutants (n = 3)

Variables				%R-TC		%R-CV		%R-MB	
A	B	C	D	Experimental	Predicted	Experimental	Predicted	Experimental	Predicted
6	21	0.014	8	91.33 ± 1.8	88.16	95.82 ± 2.5	93.25	98.19 ± 1.9	98.41

### Desorption studies

**Fig. 5:**
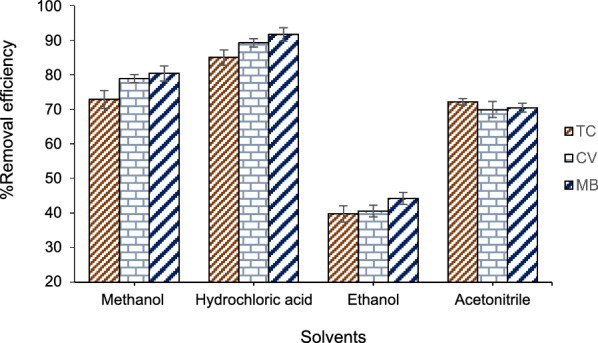
The effect of solvent on the desorption process

In the surface adsorption process, the use of an appropriate solvent for complete desorption of the adsorbed analyte from the surface of the adsorbent is essential for the reuse of the adsorbent. For desorption of analytes from the surface of the Fe_3_O_4_/rGO/Ag nanocomposite, solvents including ethanol, acetonitrile, hydrochloric acid, and methanol were investigated as desorbing agents. Figure 5 illustrates the results of this investigation and the impact of each solvent on the removal percentage of TC, CV, and MB from the adsorbent surface. According to the obtained results, hydrochloric acid is suitable for the complete desorption of TC, CV, and MB from the nano-adsorbent surface. Therefore, hydrochloric acid was employed as the desorbing solvent in subsequent experiments.

### ***The reusability of Fe***_***3***_***O***_***4***_***/rGO/Ag nanocomposite***

**Fig. 6:**
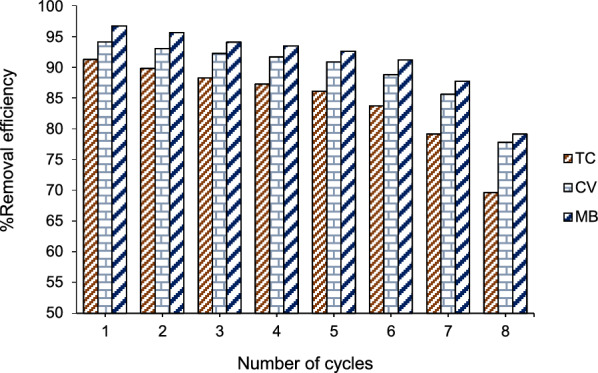
The reusability of Fe_3_O_4_/rGO/Ag nanocomposite for removal of TC, CV, and MB

The efficiency and stability of the Fe_3_O_4_/rGO/Ag magnetic adsorbent were investigated. The Fe_3_O_4_/rGO/Ag magnetic nanocomposite, employed for the removal of TC, CV, and MB, demonstrated sustained performance even after purification in two stages with 2 mL of hydrochloric acid and 2 mL of distilled water (repeated three times). The results, as depicted in Fig. 6, indicate that the magnetic adsorbent remains stable for at least 7 cycles, showing no significant reduction in analyte removal efficiency and magnetic properties.

### Real samples analysis

The proposed method for the removal of TC, CV, and MB in environmental water samples was employed to assess the method's efficiency. The studied water samples initially lacked measurable amounts of TC, CV, and MB. Therefore, specific amounts of TC, CV, and MB standard solutions were added to the samples, and the removal efficiency of the analytes was examined. The results of the water sample analysis are presented in Table 8. The obtained results indicated that the efficiency of the proposed method for TC, CV, and MB removal in water samples ranged from 87.05 to 98.08% with RSD% < 4. The achieved removal values suggest that the proposed method can be used with high accuracy for the removal of TC, CV, and MB in water samples.

**Table 8:** Results of real samples under the optimum conditions (n = 3)

Samples	Analyte	%R ± %RSD
Tap water	TC	90.88 ± 2.2
	CV	93.77 ± 3.2
	MB	97.51 ± 1.4
Wastewater	TC	87.05 ± 1.9
	CV	90.97 ± 2.4
	MB	93.81 ± 2.2
River water	TC	88.62 ± 3.0
	CV	92.86 ± 2.2
	MB	94.82 ± 3.2
Fish farm	TC	90.89 ± 3.2
	CV	95.23 ± 2.5
	MB	98.08 ± 2.3

### ***Comparison Fe***_***3***_***O***_***4***_***/rGO/Ag nanocomposite with other adsorbents***

The results indicated that Fe_3_O_4_/rGO/Ag magnetic nanocomposite could be used as high-efficiency adsorbents in removing TC, CV, and MB from different water samples. Besides, Fe_3_O_4_/rGO/Ag magnetic nanocomposite were compared with other adsorbents used to remove TC, CV, and MB in different studies (Table 9). As depicted in this table, Fe_3_O_4_/rGO/Ag magnetic nanocomposite are extremely capable of removing TC, CV, and MB in a short time with high efficiency compared to other adsorbents. Moreover, RSM reduced the number of tests, process time, and economic savings compared to other methods available in the literature.

**Table 9:** Comparison of the Fe_3_O_4_/rGO/Ag nanocomposite with other adsorbents

Adsorbent	Analyte	pH	Adsorbent amount	Time	Result	Refs.
SiO_2_ nanoparticles	TC	6	0.25 g	40 min	99.56%	[45]
Zeolitic imidazolate framework	TC	5.9	0.63 g	26.8 min	446.9 mg g^−1^	[46]
Cobalt-impregnated spent coffee ground biochar	TC	7	100 mg	25 min	370.37 mg g^−1^	[47]
Ceramsite substrate	TC	7	20 g	24 h	2.56 mg g^−1^	[48]
*Rhizophora mucronat* stem-barks	CV	7	0.25 g	60 min	99.8%	[49]
Zinc oxide nanoparticle loaded on activated carbon	CV	6	0.02 g	4 min	73.25 mg g^−1^	[50]
Alginate@silver nanoparticles	CV	7	0.01 g	240 min	186.93 mg g^−1^	[51]
Citric acid modified red-seaweed	CV	7	1.5 g	90 min	93.40%	[52]
Terminalia catappa shell	MB	5	0.1 g	45 min	90.56%	[53]
Magnetized *Tectona grandis* sawdust	MB	8	1 g	60 min	90.8%	[54]
Millet household carbon	MB	7	0.2 g	18 min	90%	[55]
Sheath palm	MB	6	30 mg	60 min	162.54 mg g^−1^	[56]
Fe_3_O_4_/rGO/Ag nanocomposite	TC	6	0.014 g	8 min	91.33%	Our work
Fe_3_O_4_/rGO/Ag nanocomposite	CV	6	0.014 g	8 min	95.82%	Our work
Fe_3_O_4_/rGO/Ag nanocomposite	MB	6	0.014 g	8 min	98.19%	Our work

## Conclusion

This study aimed to investigate TC, CV, and MB removal from water and wastewater samples by the Fe_3_O_4_/rGO/Ag magnetic nanoparticles. After the synthesis of magnetic nanocomposite, their morphology and crystalline nature were investigated by several techniques including XRD, EDX, VSM, FTIR, and FE-SEM. Investigations showed that Fe_3_O_4_/rGO/Ag magnetic nanocomposite provide high removal efficiency for TC, CV, and MB. In order to optimize the removal conditions of TC, CV, and MB, various influencing parameters such as concentration, contact time, adsorbent amount, and pH were evaluated. Modeling the removal of TC, CV, and MB using the Fe_3_O_4_/rGO/Ag magnetic nanocomposite was performed using the RSM based on the BBD. The influence of parameters on the removal process was investigated with a second-degree model. Considering the high values of R2 (0.99) and adjusted R2 (0.99), it can be concluded that the obtained model was suitable for analyzing the data of TC, CV, and MB removal by the Fe_3_O_4_/rGO/Ag magnetic nanocomposite. The optimal conditions for maximum removal of TC, CV, and MB were determined to be pH 6, a concentration of 21 mg L^−1^, a time of 8 min, and a nanocomposite amount of 0.014 g. The removal efficiencies obtained in experiments conducted at optimum conditions were 91.33, 95.82, and 98.19% for TC, CV, and MB, respectively. Also, adsorbent reusability tests showed that Fe_3_O_4_/rGO/Ag magnetic nanocomposite can be reused for more than seven cycles without significantly decreasing performance. The results demonstrated that the synthesized Fe_3_O_4_/rGO/Ag magnetic nanocomposite significantly possesses the capability to remove TC, CV, and MB from aqueous solutions in a short period. Therefore, it can be effectively utilized as an adsorbent for the removal of TC, CV, and MB. The findings also indicate that the adsorbent exhibits excellent separation ability after adsorption from the solution.

## Data Availability

The datasets used and/or analyzed during the current study are available from the corresponding author on reasonable request.
